# Comparative analysis of two optical biometry devices: high wavelength swept source OCT versus partial coherence interferometry

**DOI:** 10.1007/s10792-021-02036-0

**Published:** 2021-10-11

**Authors:** Eszter Szalai, Adrienne Csutak

**Affiliations:** Department of Ophthalmology, University of Pécs Medical School, Rákóczi u. 2, 7623 Pecs, Hungary

**Keywords:** Biometry, Partial coherence interferometry, Reproducibility, Swept source optical coherence tomography

## Abstract

**Purpose:**

To study the reproducibility of measurements performed with a recently developed multimodal high resolution swept source optical coherence tomography (SSOCT) and to make comparisons with a partial coherence interferometry (PCI) biometer.

**Methods:**

One hundred and fifty-two eyes of 152 subjects were involved in this study with a mean age of 65.71 ± 13.86 years (26–85 years). Anterior surface keratometry (K), anterior chamber depth (ACD), white-to-white (WTW) and axial length (AL) values were recorded by the SSOCT (ANTERION, Heidelberg Engineering Ltd, Germany) and PCI (IOLMaster 500, version 5.5, Carl Zeiss Meditec, Germany). Intraocular lens (IOL) power was calculated based on ANTERION and IOLMaster keratometry values by using five traditional vergence formulas.

**Results:**

Anterior surface simulated keratometry values did not differ significantly between the IOLMaster and ANTERION (*P* > 0.05). AL measurements were successful in 95% of the cases both with the SSOCT and PCI. No significant difference was disclosed between the two instruments (*P* = 0.229). For WTW measurements, a significant difference was observed between the two optical biometers (*P* < 0.0001). The difference between PCI and SSOCT in IOL powers was statistically significant for SRK/T, Hoffer and Holladay formulas (*P* < 0.001).

**Conclusion:**

Our results implicated an overall good reproducibility of anterior keratometry, AL, ACD and WTW measurements for IOLMaster and ANTERION. The discrepancies between their measurements resulted in significant difference in the calculated IOL power for SRK/T, Hoffer and Holladay formulas, but not for Haigis formula.

## Introduction

Precise calculation of the intraocular lens (IOL) is of importance in preoperative evaluation of patients with cataract. In planning the IOL power, axial length (AL) and keratometry value (K), as well as anterior chamber depth (ACD), lens thickness (LT), corneal diameter (WTW), are required depending on the applied calculation formula [1]. Partial coherence interferometry (PCI) has been the gold standard for optical biometry since its introduction in autumn 1999 [1, 2]. However, traditional PCI-based devices have not been able to provide data on the posterior corneal surface, thickness of crystalline lens and corneal pachymetry. Also, they could have difficulties in measuring dense cataracts due to the use of 780 nm wavelength laser diode infrared light. Novel third-generation optical biometers that employ swept source optical coherence tomography (SSOCT) technology include IOLMaster 700 (Carl Zeiss Meditec AG, Jena, Germany), Argos (Movu, Inc., CA, USA), OA-2000 (Tomey, Nagoya, Japan), Eyestar 900 (Haag Streit, Koeniz, Switzerland) and ANTERION (Heidelberg Engineering, Germany). These instruments use longer wavelengths ranging from 1050 to 1300 nm that allow for less scattering and deeper penetration through opaque media.

Traditional keratometry instruments measure radius of curvature of the paracentral anterior cornea and calculate corneal power using a standard keratometric index. Hypothetically, determination of total corneal power calculated based on both anterior and posterior corneal curvatures should improve the accuracy in IOL power prediction. Anterior segment OCTs can measure both anterior and posterior corneal curvatures with high axial resolution to compute total corneal power.

The aim of this study was to investigate the reproducibility of measurements performed using a recently introduced multimodal high resolution swept source OCT and to make comparisons with a traditional PCI biometer. We also investigated the impact of discrepancy between the two optical biometers on IOL calculation using different traditional formulas.

## Materials and methods

### Study design

Ocular biometry was performed in 152 phakic eyes of 152 patients (98 females, 54 males; 82 right, 70 left eyes) using a newly developed high wavelength swept source anterior segment OCT and PCI (Zeiss IOLMaster version 5.5, Carl Zeiss Meditec AG, Jena, Germany). The mean age was 65.71 ± 13.86 years (ranging from 26 to 85 years). All subjects had a negative history of ocular disease (other than refractive errors excluding corneal ectasias), trauma or surgery. The study population included a wide spectrum of disease severity, with the vast majority of patients graded clinically as moderate to advanced lens opacities. Five percent of study patients had intumescent lenses and only 12% had low grade changes. The local Institutional Review Board approved this study in accordance with the Declaration of Helsinki.

ANTERION® SSOCT employs a 1300 nm light source and its Cataract App (Heyex Software, Version 2.4.3, Heidelberg Engineering) performs biometry analysis which combines important measurements for preoperative IOL planning including comprehensive corneal assessment, aqueous depth (AQD), lens thickness, WTW and axial length. ACD was calculated by adding the CCT to the AQD. Comprehensive corneal assessment is achieved by measuring the anterior and posterior cornea and computing multiple maps (anterior and posterior axial curvature, tangential curvature and elevation maps, as well as total corneal power map, anterior and total corneal wave front and pachymetry maps). SSOCT computes total corneal power based on measuring the anterior and posterior corneal surface in a central 3 mm ring, total K is defined as the average refractive power of the cornea, derived from the anterior and posterior corneal surfaces. The conversion of anterior radii to keratometry values is performed according to the laws of Gaussian optics using the keratometric index of 1.3375. Anterior axial simulated keratometry values are calculated with the indicated keratometric index for a 3 mm ring. IOLMaster utilizes PCI to evaluate axial length, based on the Michelson interferometer. Keratometry values of the IOLMaster are obtained from six points reflected off the anterior surface of the central cornea (approximately in a 2.5 mm diameter). The IOLMaster uses a slit beam of light through the anterior segment of the eye at and the internal software measures the distance between the anterior corneal surface and the anterior crystalline lens surface to calculate ACD [3]. For WTW measurements, the same principle was applied by the IOLMaster.

Biometry measurements by SSOCT and PCI were taken on each eye in random order. Patients were positioned on the forehead and chin rest, and they were instructed to fixate on the built-in fixation light of the instrument. Biometry measurements by SSOCT and PCI were taken on each eye in random order. Anterior segment biometry parameters and instrument-based IOL power calculations were recorded and used for further analysis.

Optical biometry readings of IOLMaster and ANTERION were used to predict the IOL power with four paraxial thin lens IOL calculation formulas. The Hoffer Q, Holladay I, Haigis and SRK/T formulas were applied and analyzed [4–6]. For IOLMaster and ANTERION, simulated keratometry of the anterior corneal surface was used. In every case, IOL power calculations were performed using the IOL constants for Acrysof SN60WF (Alcon Laboratories Inc.) (available at http://ocusoft.de/ulib/c1.htm).

### Statistical analysis

Statistical analyses were performed with SPSS (Version 9.0.0) and MedCalc Statistical Software (Version 10.0.2.0). Data were described as mean ± standard deviation (SD) and 95% confidence intervals (CI) were also provided. The Kolmogorov–Smirnov test was used to analyze whether our data were normally distributed. To determine the difference between two measurements, Student’s *t*-test was applied. The correlation between instruments was calculated by Pearson’s correlation test. Bland–Altman plots were created, and the 95% limits of agreement (95% LoA = mean difference ± 1.96 SD of the difference) were determined to compare two methods [7, 8]. To estimate intra-device consistency, intraclass correlation coefficients (ICC) and their 95% CI were calculated. The results were defined as statistically significant if *P* value was less than 0.05.

## Results

**Fig. 1:**
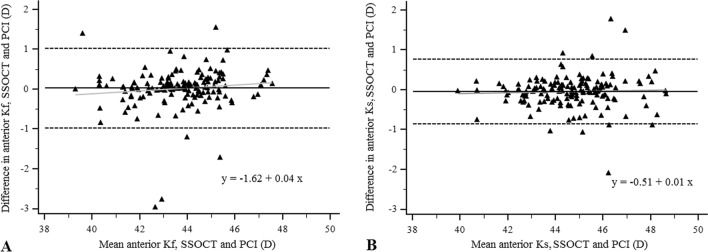
Bland–Altman plots of the difference in anterior flat keratometry (Kf) and in anterior steep keratometry (Ks) measurements between ANTERION (SSOCT) and IOLMaster (PCI) against their mean (**A**, **B**, respectively)

All biometry measurements obtained by both devices are summarized in Table 1. Anterior surface simulated keratometry values did not differ significantly between the IOLMaster and ANTERION. High reproducibility was found in Ks and Kf values for the two instruments (ICC = 0.951 and 0.970, respectively). Bland–Altman plots showed low difference with acceptable 95% LoA for the keratometry values (Fig. 1). Total Kf was 42.94 ± 7.73 D (95% CI: 41.70–44.19 D), total Ks was 43.51 ± 6.06 D (95% CI: 42.53–44.48 D) measured with SSOCT. Both the total Kf and total Ks were lower than the simulated Kf (*P* = 0.223) and Ks, but the difference (Kf: − 0.74 ± 7.44 D; Ks: − 1.14 ± 5.91 D) was only significant for Ks (*P* = 0.019). The correlation was poor both between simulated Kf and total Kf (*r* = 0.279, *P* = 0.0005), and between simulated Ks and total Ks (*r* = 0.231, *P* = 0.004) obtained with SSOCT. The magnitude of anterior surface astigmatism did not differ significantly between the two instruments (*P* = 0.182). Good intra-device consistency was indicated by ICC (0.823) and small difference was obtained between the SSOCT and PCI for anterior surface astigmatism.

**Table 1:** Anterior corneal parameters and biometry values measured with partial coherence interferometry (PCI) and swept source optical coherence tomography (SSOCT)

	PCI^a^	SSOCT^a^	P*	*r*^#^	ICC (95% CI)	Difference (SSOCT–PCI)	95% LoA
Kf (D)	43.67 ± 1.60 (43.41–43.92)	43.68 ± 1.67 (43.42–43.95)	0.609	0.952 (< 0.0001)	0.951 (0.934–0.965)	+ 0.02 ± 0.51	− 0.98 to + 1.02
Ks (D)	44.69 ± 1.67 (44.42–44.96)	44.64 ± 1.697 (44.37–44.92)	0.189	0.970 (< 0.0001)	0.970 (0.959–0.978)	− 0.04 ± 0.41	− 0.86 to + 0.77
Astigmatism (D)	1.02 ± 0.829 (0.88–1.15)	0.96 ± 0.85 (0.83–1.10)	0.182	0.824 (< 0.0001)	0.823 (0.764–0.869)	− 0.06 ± 0.50	− 1.17 to + 0.93
Cylinder axis (Degree)	87.72 ± 60.89 (77.92–97.51)	85.97 ± 45.58 (78.64–93.30)	0.776	− 0.310 (< 0.0001)	− 0.298 (− 0.437–− 0.145)	− 2.02 ± 86.73	− 172.01 to + 167.97
AL (mm)	23.33 ± 1.25 (23.13–23.54)	23.24 ± 1.12 (23.05–23.42)	0.229	0.837 (< 0.0001)	0.832 (0.774—0.877)	− 0.07 ± 0.69	− 1.43 to + 1.29
ACD (mm)	3.19 ± 0.388 (3.13–3.25)	3.18 ± 0.395 (3.12–3.24)	0.880	0.914 (< 0.0001)	0.914 (0.882–0.937)	− 0.002 ± 0.16	− 0.32 to + 0.32
WTW (mm)	12.00 ± 0.46 (11.92–12.08)	11.69 ± 0.45 (11.61–11.76)	< 0.0001	0.768 (< 0.0001)	0.768 (0.690–0.828)	− 0.32 ± 0.31	− 0.93 to + 0.30

*LoA* limits of agreement

*Paired *t*-test (PCI versus SSOCT)

^#^Pearson’s correlation coefficient

^a^Mean ± SD (95% confidence interval)

**Fig. 2:**
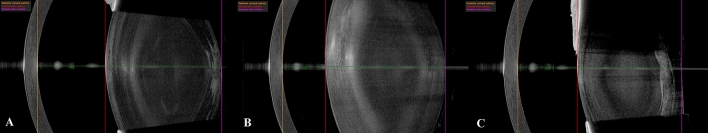
Visualization of the severity of lens opacities with the ANTERION Cataract App. **a** Posterior subcapsular cataract and dense nuclear opacities, neither ANTERION nor IOLMaster was able to perform axial length (AL) measurement. **b** Anterior subcapsular and dense nuclear cataract, IOLMaster was not able to measure AL. **c** Posterior subcapsular cataract, ANTERION was not able to identify posterior lens surface

**Fig. 3:**
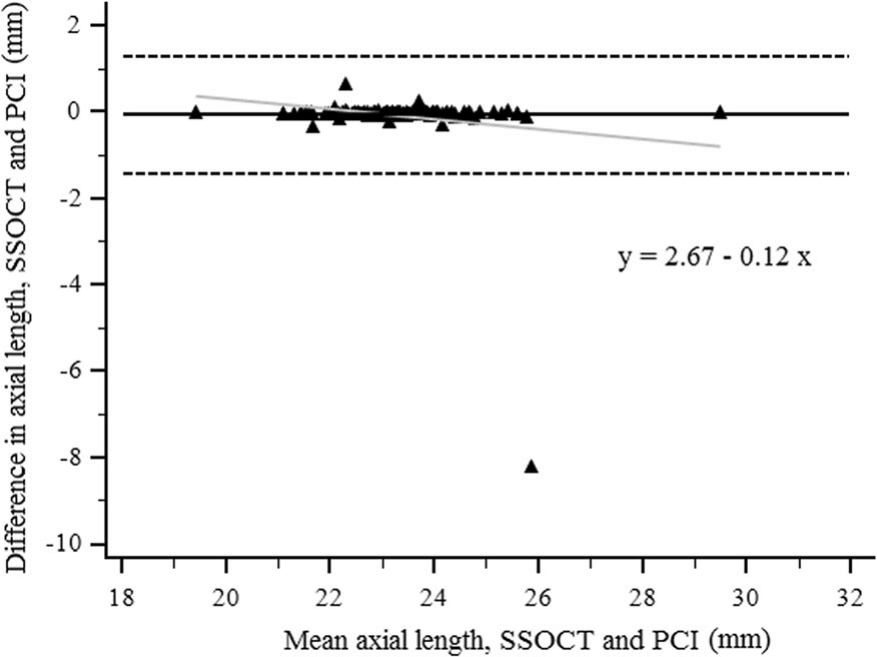
Bland–Altman plots of the difference in axial length measurements between ANTERION (SSOCT) and IOLMaster (PCI) against their mean

Axial length measurements were successful in 95% of the cases (145/152) both with the SSOCT and PCI; in 3 cases (2%) neither of the two devices was able to measure AL (Fig. 2). No significant difference was disclosed between the two instruments (*P* = 0.229) and good reproducibility was observed for them in the AL measurements (ICC = 0.832). Bland–Altman analysis indicated low difference between the instruments with wide 95% LoA (Fig. 3).

AQD measured with ANTERION was 2.64 ± 0.395 mm (95% CI: 2.57–2.70 mm). ACD measurements were not significantly different between the two instruments (*P* = 0.880). ICC indicated high reproducibility for the instruments (0.914) and the difference was low (0.002 ± 0.16 mm) with narrow 95% LoA.

**Fig. 4:**
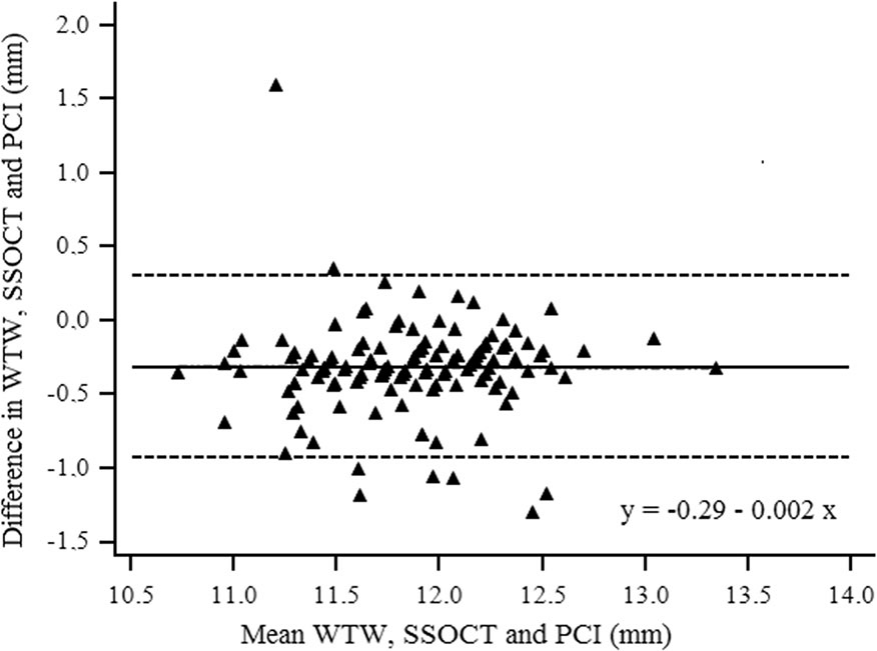
Bland–Altman plots of the difference in white-to-white (WTW) measurements between ANTERION (SSOCT) and IOLMaster (PCI) against their mean

For WTW measurements, statistically significant difference was observed between the two optical biometers (*P* < 0.0001). The reproducibility of both devices was acceptable (ICC = 0.768) in measuring WTW. Bland–Altman plot showed small difference in WTW values between SSOCT and PCI with narrow 95% LoA (Fig. 4).

**Fig. 5:**
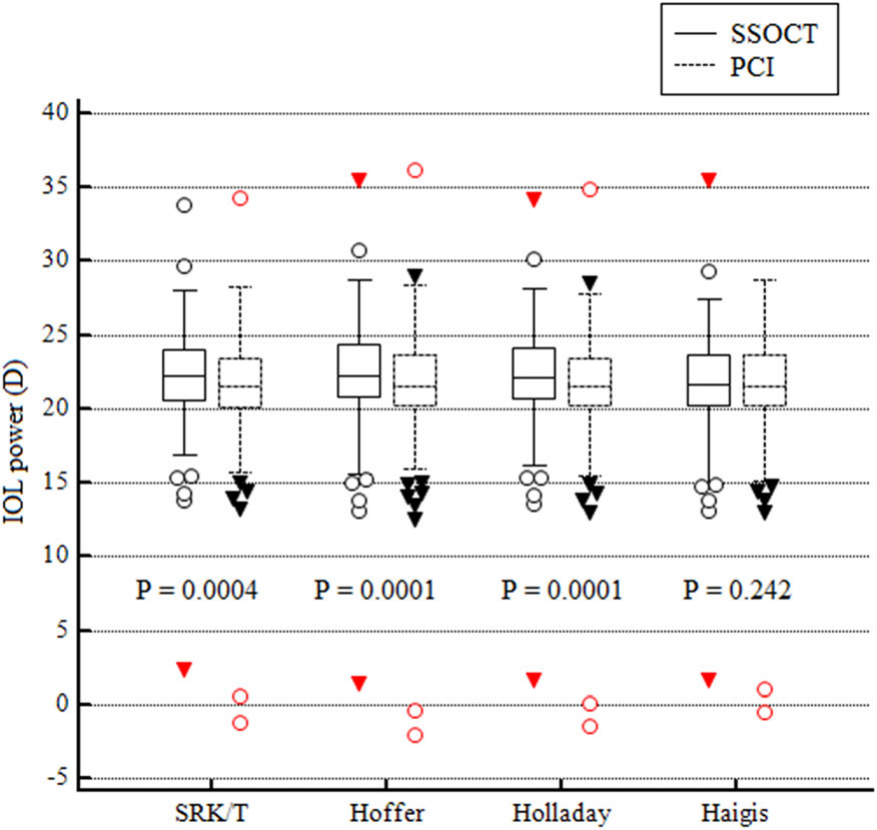
Comparison of intraocular lens (IOL) power computed with four traditional calculation formulas using anterior simulated keratometry measured by ANTERION (SSOCT) and IOLMaster (PCI). The difference between their evaluations (*P* value) was calculated with Student’s *t*-test

For all IOL calculation formulas, SSOCT using simulated keratometry of the anterior corneal surface resulted in higher IOL power than that for PCI (Fig. 5). The difference between PCI and SSOCT in IOL powers was statistically significant for SRK/T, Hoffer Q and Holladay I formulas (*P* = 0.0001–0.0004), but not for Haigis formula (*P* = 0.242) (Fig. 5).

## Discussion

Swept source OCT is a recent development of Fourier domain OCT using a tunable laser as a light source [9]. There are several advantages of a swept source OCT technique over the former systems, including short image acquisition time, less motion artifacts, high axial, lateral resolution, deeper tissue penetration, improved safety profile [10]. During biometry, a total of 65 radial B-scans (256 A-scans per B-scan) are acquired using the ANTERION Cornea App and one anterior segment biometry evaluation (768 A-scans per B-scan) is performed in the Cataract App. Although not able to quantify the severity of cataract, recent SSOCT devices are able to provide a subjective estimation of the severity of opacities and visualization of the crystalline lens.

ANTERION is capable of performing a comprehensive corneal analysis and the result is integrated in its Cataract App. In this study, anterior surface simulated keratometry values did not differ significantly between the IOLMaster and ANTERION. Yang et al. reported no significant difference in average simulated keratometry value between PCI and another SSOCT device (Movu Argos) [11]. Total corneal power can be calculated from the anterior corneal radius and this estimation should theoretically be identical to the total corneal power measured by three dimensional tomographers [12]. Interestingly, poor correlation was observed both between simulated Kf and total Kf and between simulated Ks and total Ks measured with ANTERION in this study.

New SSOCT biometers are thought to be more often successful at evaluating axial length in opaque cataracts based on their optical principles [13]. In our study, AL measurements were successful in 95% of the cases both with SSOCT and PCI. Previous authors demonstrated high success rate of AL evaluation with different SSOCT devices ranging from 92.5 to 100% depending on the severity of lens opacities [11, 14–19]. In contrast to our findings, recent articles described a failure rate of PCI in measuring AL between 31 and 38% [19, 20]. In accordance with other authors [11, 17, 19, 21, 22], we did not observe a significant difference in AL values between SSOCT and PCI and ICC indicated good intra-device reproducibility for their measurements.

In cataract surgery, surgical outcome could be affected by the precise ACD measurements through the IOL power calculation and determination of the effective postoperative IOL position [23]. ACD is variously defined as the distance between the corneal epithelium and the anterior surface of the lens (external ACD), and as the distance between the corneal endothelium and the lens (internal ACD). ACD is measured along the visual axis from the corneal epithelium to the anterior crystalline lens by using IOLMaster 500, thus it includes the thickness of the cornea. ANTERION (Software Version 2.4.3) displays aqueous depth that is defined as the distance from the posterior corneal surface to the anterior lens surface. The external ACD could be calculated by adding the CCT to the AQD which might result in some sources of error. Consistent with the results of previous studies, ACD measured by a PCI device was deeper than that obtained by a SSOCT device but the difference was not statistically significant [11, 15, 24]. Yang et al. attributed this fundamental discrepancy in ACD measurements to the distinct imaging principles of PCI devices and SSOCTs [11]. Fisus et al. recently observed a mean absolute difference of 0.07 ± 0.04 mm for ACD between the ANTERION and IOLMaster 700 [25].

Corneal diameter is important in determining the accurate size of phakic IOLs [26]. The IOLMaster evaluates the horizontal WTW distance automatically, the ANTERION offers a high resolution 16 mm WTW scan and computes the WTW value in the horizontal meridian. We observed a statistically significant difference in WTW measurements between PCI and SSOCT, but the intra-device consistency was acceptable. Agreeing with our previous study, PCI significantly overestimated WTW when compared to anterior segment OCT [26]. Other previous work concluded that WTW measurements with different devices suffer from significant inaccuracies [27, 28]. For anterior chamber angle supported IOL sizing, angle-to-angle distance would be more useful; this can be measured with OCT. For posterior chamber IOL sizing, most studies have used WTW plus 0.5 mm [29]; although sulcus-to-sulcus diameter would be more precise.

Another aim of this study was to investigate the impact of discrepancies between PCI and SSOCT on IOL calculation using different traditional formulas. All of these formulas are paraxial thin lens vergence formulae; they differ in the type of biometry parameters necessary to calculate IOL power [30]. Besides anterior axial curvature, total corneal power by ray tracing is also included in the ANTERION Cataract App. Savini et al. investigated the difference between total corneal power and simulated keratometry values provided by a Scheimpflug camera combined with Placido-disk topographer and their impact on accuracy of IOL power calculation [31].They assumed that direct calculation of total corneal power (instead of estimation of simulated keratometry) might improve the accuracy of IOL power calculation [31]. Previous authors concluded that using total corneal power calculated with Pentacam did not result in significantly lower IOL prediction error [12, 31]. We did not find a significant difference in computed IOL power using Haigis formula between IOLMaster and ANTERION. It should be noted that since the aim of this study was to compare the traditional PCI and the most recent swept source OCT biometer on a general population, the authors employed commonly used third-generation formulae, Holladay 1, SRK/T and Hoffer Q, as well as a fourth-generation formula, Haigis which requires ACD to estimate ELP [4–6]. The authors assume that the advantages of SSOCT in measuring total corneal power and lens thickness might be utilized better by using newer generation formulas in predicting IOL power more precisely.

In conclusion, our results implicated an overall good reproducibility of IOLMaster 500 and ANTERION in anterior keratometry, AL, ACD and WTW measurements. Both biometry devices had high success rate in evaluating axial length. The discrepancies between their measurements resulted in a significant difference in the calculated IOL power using SRK/T, Hoffer Q and Holladay 1 paraxial thin lens vergence formulas, but not for Haigis formula. Further studies are needed to investigate the repeatability and reliability performance of ANTERION SSOCT in biometry measurements.

## Data Availability

Data are available upon request.
